# Past, present, and future of cell replacement therapy for parkinson’s disease: a novel emphasis on host immune responses

**DOI:** 10.1038/s41422-024-00971-y

**Published:** 2024-05-22

**Authors:** Tae-Yoon Park, Jeha Jeon, Young Cha, Kwang-Soo Kim

**Affiliations:** 1 Molecular Neurobiology Laboratory, Department of Psychiatry and McLean Hospital, Harvard Medical School, Belmont, MA USA; 2 Program in Neuroscience, Harvard Medical School, Belmont, MA USA; 3 Department of Neurosurgery, Massachusetts General Hospital, Harvard Medical School, Boston, MA USA; 4 Harvard Stem Cell Institute, Harvard Medical School, Belmont, MA USA

**Keywords:** Induced pluripotent stem cells, Immunology

## Abstract

Parkinson’s disease (PD) stands as the second most common neurodegenerative disorder after Alzheimer’s disease, and its prevalence continues to rise with the aging global population. Central to the pathophysiology of PD is the specific degeneration of midbrain dopamine neurons (mDANs) in the substantia nigra. Consequently, cell replacement therapy (CRT) has emerged as a promising treatment approach, initially supported by various open-label clinical studies employing fetal ventral mesencephalic (fVM) cells. Despite the initial favorable results, fVM cell therapy has intrinsic and logistical limitations that hinder its transition to a standard treatment for PD. Recent efforts in the field of cell therapy have shifted its focus towards the utilization of human pluripotent stem cells, including human embryonic stem cells and induced pluripotent stem cells, to surmount existing challenges. However, regardless of the transplantable cell sources (e.g., xenogeneic, allogeneic, or autologous), the poor and variable survival of implanted dopamine cells remains a major obstacle. Emerging evidence highlights the pivotal role of host immune responses following transplantation in influencing the survival of implanted mDANs, underscoring an important area for further research. In this comprehensive review, building upon insights derived from previous fVM transplantation studies, we delve into the functional ramifications of host immune responses on the survival and efficacy of grafted dopamine cells. Furthermore, we explore potential strategic approaches to modulate the host immune response, ultimately aiming for optimal outcomes in future clinical applications of CRT for PD.

## Introduction

Parkinson’s disease (PD) was first described as ‘shaking palsy’ by Dr. James Parkinson in 1917.^1^ Since then, numerous scientists have investigated the pathological mechanisms of this common yet incurable neurodegenerative disorder. Despite substantial efforts, the precise mechanisms underlying the pathogenesis of PD remain elusive, with no treatment available yet that can slow down, stop, or reverse its progression.^2–5^ However, these studies have consistently highlighted that degeneration of a specific cell type, midbrain dopamine neurons (mDANs) in the substantia nigra, is the major pathological feature associated with PD’s characteristic motor deficits, such as bradykinesia, rigidity, tremor, and postural instability.^2–5^

This observation has led many scientists to explore cell replacement therapy (CRT) as a potential treatment for PD, by replacing the lost mDANs. Starting from the 1980s, various types of dopamine-producing cells have been tested in both pre-clinical and clinical studies, including retinal cells, adrenal medullary cells, carotid body cells, and human and porcine fetal cells.^6–13^ Among these, a groundbreaking study by Lindvall, Björklund, and their colleagues involved the transplantation of dopamine-rich tissues dissected from fetal ventral mesencephalon (fVM) into a PD patient, resulting in substantial recovery and providing the first “proof-of-concept” for CRT.^14^ Subsequent open-label studies reported significant and long-term improvements in some patients.^15–31^ However, fVM-based CRT faced numerous challenges, leading current translational stem cell research to focus on characterizing human pluripotent stem cells (hPSCs), such as human embryonic stem cells (hESCs) and induced pluripotent stem cells (hiPSCs), as potential sources of transplantable midbrain dopamine (mDA) cells.^6–13^

Although fVM transplantation revealed serious issues, it provided not only a “proof-of-concept” but also invaluable insights and lessons for the future optimization of CRT. A compelling question arises: Why were the clinical outcomes of previous fVM transplantation highly variable and inefficient? In this review, we will discuss important lessons that can be learned from previous fVM transplantation studies and address critical issues necessary for the success of hPSC-based CRT. Drawing on recent findings from our laboratory and others’, we will highlight host immune responses as major risk factors that limit the survival and maturation of the grafted mDA cells, as well as the overall clinical outcomes of CRT in PD. Additionally, we will explore potential strategic approaches to modify the host immune response and other factors for optimal outcomes in future clinical applications of CRT.

## Lessons from FVM transplantation studies

### Clinical outcomes of fVM-based CRT varied widely, occasionally accompanied by graft-induced dyskinesia (GID)

Starting from the late 1980s, stem cell scientists attempted CRT using fVM tissues dissected from aborted embryos (typically 6 to 9 weeks old) because they showed the most promising outcomes among various dopamine-producing cells.^6–13^ In these early open-label trials using fVM tissues, some patients showed prominent and long-term improvement including enhanced movement, reduced rigidity and tremor; decreased ‘off’ periods; reduction of medication, or in a few cases, a complete cessation; and enhanced dopamine uptake, as evidenced by ^18^F-DOPA PET scan analyses (Table 1).^14–35^ Subsequent postmortem studies of deceased transplanted patients further supported these positive clinical outcomes, revealing successful engraftment of implanted mDANs with robust outgrowth and innervation to the host striatum, demonstrating that engrafted mDANs were functionally integrated into neural circuits in the patient’s brain. In summary, these successful clinical data provided the “proof-of-concept” of CRT in PD. Despite these positive results, subsequent double-blind, sham-controlled studies showed clinical benefits were statistically insignificant and this method was not recommended as a treatment for PD.^36–38^ Furthermore, ∼30% of transplanted patients (18 out of 56) developed a serious side effect: GID,^36–38^ significantly dampening the initial enthusiasm for the fetal cell-based approach. These inconsistent and disappointing outcomes, along with ethical, medical, and practical limitations, rendered this approach unfeasible as a standard treatment for PD.^6–11^

**Table 1:** List of representative clinical trials using human fetal tissues.

Number of patients	Average age of cohort (range)	Disease duration (years)	Number of fVM/side (age)	Grafted site	Immuno-suppression (period)	Follow-up period	TH^+^ cell survival	Outcome	Adverse effect	Ref.
2	51.5 (48–55)	7	3–4 (8–10 wks)	Unilateral (C+P)	CsA, AZA, PSL	2 yrs	N/A	Minor improvement of motor speed for pronation-supination	None	^14,24^
2	55 (50–60)	8–11	4 (6–7 wks)	Unilateral (P)	CsA, AZA, PSL (64 mo)	10 yrs	*n* = 1 6246 (rostral); 10,728 (middle); 25,854 (caudal)	Improved rigidity and bradykinesia	None	^25,26^
7	56.3 (39–66)	7–20	1 (7–8 wks)	2 pts-unilateral(C+P); 5 pts-bilateral (P)	CsA, PSL in 4 pts	1–4 yrs	N/A	Motor improvement; Shorter and less severe dyskinesia	None	^18^
2	36.5 (30–43)	6	3–4 (6–8 wks)	Bilateral (C+P)	CsA (12 mo), AZA (18 mo), PSL (N/A)	2 yrs	N/A	Improved motor functions	None	^31^
4	52 (47–63)	10–21	1 (7–11 wks)	Unilateral (C)	CsA (6 mo)	1.5 yrs	N/A	Improved motor functions (3/4)	Transient panic disorder (1/4)	^29^
2	56 (49–63)	10–17	2–3 (6–9 wks)	1 pt–unilateral (P); 1 pt–bilateral (C+P)	CsA (7 mo), AZA, PSL	1.5 yrs	N/A	Improved motor functions	Transient obsessive compulsive disorder (1/2)	^17^
4	52.3 (39–61)	8–22	3–4 (6.5–9 wks)	Bilateral (P)	CsA (6 mo)	1.5 yrs	Patient 1 (18 mo): 128,162 (right), 81,905 (left); Patient 2 (19 mo): 79,207 (right), 39,151 (left)	Improved motor functions; Resolution of dyskinesias	Cortical hemorrhage (1/4); Transient confusion and hallucinations (1/4)	^19,22,23^
5	57.4 (48–67)	13–22	1–3 (6–9 wks)	1 pt–unilateral (P); 4 pts–unilateral (C+P)	CsA (7 mo), AZA, PSL	3 yrs	N/A	Modest improvement in daily living activities (1/5)	None	^15^
6	48.8 (43–58)	7–16	4–8 (6–8 wks)	4 pts-unilateral (P); 1 pt-unilateral (C+P)	CsA, AZA, PSL	${{{{{\rm{\le }}}}}}$18 yrs	N/A	Major improvement (4/6); Modest changes (2/6)	Atypical parkinsonism (1/6); Dementia (1/6)	^20,21,30^
20	$\le$ 60 yrs: 50 ± 8; > 60 yrs: 65 ± 4	$\le$ 60: 13 ± 3; > 60: 15 ± 6	2 (7–8 wks)	Bilateral (P)	None	3 yrs	Patient 1 (7 mo): 24,115 (right), 38,392 (left); Patient 2 (36 mo): 36,796 (right), 6840 (left)	Improved UPDRS score in younger pts($\le$ 60 yrs), but not in older pts(> 60 yrs)	Dystonia and dyskinesia (5/20)	^37^
3	53 (48–59)	10–14	6 (6–9 wks)	Bilateral (P+SN)	CsA (6 mo)	1.1 yrs	N/A	Improved UPDRS score; Decreased L-DOPA dose requirement (1/3)	None	^16^
23	58.5 (30–75)	11	1–4 (6–9 wks)	Bilateral (P)	CsA (6 mo)	2 yrs	1 fVM (*n* = 2): 30k/side; 4 fVM (*n* = 2): 70k-120k/side	No improvement	Dyskinesia (13/23)	^38^
2	64 (59–69)	11–15	2–3 (6–9 wks)	Bilateral (P)	CsA (6 mo)	3 yrs	Patient 1 (44 mo): 127,189 (right), 98,913 (left); Patient 2 (52 mo): 202,933 (right), 4289 (left)	Improved UPDRS and Dyskinesia scores	None	^27^

*fVM* fetal ventral mesencephalic, *yrs* years, *wks* weeks, *mo* months, *C* Caudate, *P* Putamen, *SN* Substantia Nigra, *CsA* cyclosporin A, *AZA* Azathioprine, *PSL* Prednisolone, *pts* patients, *N/A* not available, *UPDRS* Unified Parkinson’s Disease Rating Scale, *Ref.* Reference.

### The poor and variable survival rates of implanted mDANs may underlie the inconsistent clinical outcomes observed in fVM-based CRT

Why were clinical outcomes of fVM transplantation inefficient and variable? Over the last several decades, more than 400 PD patients have undergone fVM transplantation and have been thoroughly analyzed.^6–13^ Additionally, stem cell scientists conducted extensive pre-clinical studies using rat and mouse embryonic VM tissues with three objectives: (1) to understand inefficient and variable clinical outcomes of fVM transplantation; (2) to elucidate the biological factors influencing the survival of transplanted cells; and (3) to enhance the survival of implanted mDANs for future successful CRTs.^39–41^ These clinical and pre-clinical studies revealed important insights and diverse potential factors contributing to inconsistent clinical outcomes. These factors encompass a range of variables, including the differing ages and clinical statuses of patients; varied fetal cell preparation methods; the heterogenous and variable status of fetuses (typically requiring 6 to 8 fetuses per patient); variable immune suppression regimens; and target sites of transplantation (putamen, caudate, and/or the substantia nigra) (Table 1).^6–14,18–31,35,37,38,42^ While all these donor- and/or host-specific factors likely play important roles in clinical outcomes, we speculate that the poor and variable survival of transplanted mDANs in the graft directly causes inefficient clinical outcomes. Indeed, this survival issue of grafted mDANs has been a focal point of CRT research since the conception of fVM transplantation due to the limited supply of aborted fetuses. As outlined in an insightful review by Brundin and colleagues,^43^ the survival of grafted mDANs in 31 independent studies using rat embryonic VM transplantation ranged between 0.7% and 23.3% with an average of 6.86%. Similarly, a consistent survival range between 5% and 10% was reported in transplantation studies of human VM grafts in athymic rats^44,45^ as well as in postmortem studies of human fVM transplantation.^9,12,13,22,23,27,37,38,42^ In conclusion, the pivotal lesson from previous clinical and pre-clinical studies is the highly inefficient and variable survival of grafted mDANs, which likely constitutes the root cause of the poor and inconsistent clinical outcomes observed.

### When and why do grafted mDANs die during fVM transplantation?

As described above, the survival of transplanted mDANs was extensively investigated in pre-clinical studies using rodent embryonic VM cells to model human fVM transplantation.^43,46–52^ A salient feature of these studies is that a great majority of mDANs die shortly after transplantation, typically within one week, and the number of surviving mDANs in the graft does not increase or change at later time points. This observation is rather surprising considering that embryonic VM cells contain a significant number of early progenitor cells in addition to already differentiated mDANs. These data suggest two possibilities: (1) progenitor cells in fVM have very limited capacity for proliferation and differentiation and/or (2) the host environment does not support their proliferation and differentiation into mDANs. Based on these pre-clinical studies, four distinct phases have been proposed during which grafted mDANs may die.^43^ In Phase 1 (removal of embryos), mDANs may die of hypoxic and hypoglycemic insults that occur during embryo removal from the maternal blood supply. In Phase 2 (cell preparation), mDANs may die of axotomy and other traumatic damages caused by mechanical dissociation. In Phase 3 (intrastriatal injection), mDANs may die during the implantation procedure and the immediate period following graft injection, and in Phase 4 (graft maturation), mDANs may die during maturation and innervation in the host brain.

Extensive studies have been conducted to understand the poor survival of grafted mDANs and the reasons for their early death post-transplantation, revealing diverse possibilities. When mDANs, dissected from embryos, are transplanted into the striatum under PD conditions, they become deprived of nutrients and growth factors crucial for their sustenance. Aligning with this concept, pre-treatment of cells with growth factors like basic fibroblast growth factor (bFGF) and glial cell line-derived neurotrophic factor (GDNF) has significantly enhanced the survival of grafted mDANs. Notably, while bFGF pre-treatment resulted in an approximately 2-fold increase in survival rate,^53,54^ continuous delivery through co-transplantation of bFGF-overexpressing fibroblasts led to a 10-fold increase, fostering more robust and rapid behavioral recovery.^55^ In addition, since neuronal injury is often associated with excitotoxicity, oxidative stress, and calcium imbalance, researchers have explored pathways and molecules associated with these phenomena. Among these, calcium channel blockers (such as flunarizine) and lipid peroxidation inhibitors (like lazaroids) have shown notable effects on mDAN survival.^56–58^ Additionally, inhibition of cell death pathways, such as caspase inhibitors (e.g., Ac-YVAD-cmk) significantly enhanced mDAN survival.^51^

## Critical issues for successful and reliable CRT using HPSC-Derived mDA cells for PD

Recognizing the insurmountable obstacles presented by fVM-based CRT, scientists have shifted their focus to the use of hPSCs, including hESCs and hiPSCs. One of the most important advantages of hPSCs is their indefinite self-renewal and pluripotent differentiation potential, enabling the production of unlimited amounts of transplantable cell sources. Although invaluable lessons and insights are available from previous fVM transplantation studies, it is evident that dopamine cells from fVM and hPSCs differ significantly in many aspects. While hESCs and hiPSCs share similarities, they also retain significant differences, in particular regarding their immune responses to the host.^59^ For instance, hESCs are inherently allogeneic and offer the advantage of being a single, standard “off the shelf” cell source, which saves time and expense. However, they require immunosuppression and are typically passaged many times, increasing the risk of harmful mutations.^60^ In contrast, autologous hiPSCs are derived from PD patients being treated, without the need for immunosuppression^61^ and would be available at earlier passages with less genetic burdens.

### Safety of hPSC-based CRT

Addressing the potential for tumor formation: the risk of tumor formation represents the most significant concern in any CRT utilizing hPSCs.^62^ Notably, there has not been a single case of tumor formation reported in fVM-based CRT among over 400 transplanted PD patients, nor in numerous embryonic VM-based pre-clinical studies.^6–13^ Currently, most CRT approaches favor the use of hPSCs, owing to their unlimited capacity for proliferation and differentiation.^63^ Ironically, this distinct advantage of hPSCs also presents a critical challenge due to their potential for neoplastic growth. In light of this, several important aspects must be considered for the clinical application of hPSC-based CRT.

Firstly, given their tumorigenic potential, it is crucial to remove any residual undifferentiated hPSCs from the transplantable cell source. For example, if merely 0.01% of cells remain undifferentiated during in vitro differentiation, this would theoretically result in 1000 undifferentiated hPSCs in a batch of 10 million cells used for transplantation. Therefore, the complete removal of any undifferentiated cells is imperative. A promising strategy for achieving this goal involves using small molecules that can selectively target and eliminate undifferentiated hPSCs without affecting differentiated cells. For example, Lee et al.^64^ discovered that undifferentiated hPSCs selectively express the anti-apoptotic gene *BIRC5* (encoding Survivin), unlike their differentiated counterparts. Based on this finding, a chemical method was developed using quercetin (a *BIRC5* inhibitor) which can eliminate undifferentiated hPSCs with > 99.99% efficiency.^65^ Using qRT-PCR analysis of *OCT4* expression as a surrogate marker, the estimated presence of undifferentiated cells post-quercetin treatment is approximately 0.0017 per 10 million differentiated cells,^65^ a rate lower than the spontaneous incidence of glioma.^66^ This chemical method was successfully used in the first hiPSC-based CRT for a sporadic PD patient.^61^

Secondly, ensuring the genomic and epigenomic stability of hPSC-derived cell products is critical. It is well known that hPSCs contain and accumulate diverse mutations of various sizes (from point mutations to karyotype abnormality).^67,68^ These mutations may have different origins, including pre-existing somatic mutations in original cells used for reprogramming,^69–73^ the reprogramming process to generate hiPSCs,^71,74–76^ passaging,^71,77^ and in vitro differentiation process^78^ to produce transplantable cell source. Various technologies such as whole genome and exome sequencing, bulk RNA-seqencing (RNA-seq) and single-cell RNA-seq analyses utilizing next-generation sequencing technology, have been employed to screen for genomic mutations and assess their functional consequences. Significantly, Merkle et al. conducted screenings on multiple hESC lines, discovering five of these lines, including the widely used H9, carry six mosaic mutations in *TP53*, which could confer a potential growth advantage.^60^ Therefore, periodic monitoring of putative tumorigenic mutations in hPSCs and their cell products is essential for their clinical application.^79^

Finally, the immunogenicity of hPSC-derived cell products is a crucial consideration in their clinical application. While iPSC-derived cells are generally believed to exhibit "negligible" immunogenicity,^80–82^ the emergence of aberrant immunogenic antigens during the differentiation process can induce immune responses.^83^ Therefore, there has been a recent emphasis on periodically monitoring potential immunogenic factors in hPSCs and their derivatives to ensure their suitability for clinical use. This includes assessing the expression levels of immunogenic-related genes such as human leukocyte antigens (*HLAs*), *CD80/CD86/CD40*, and *PD-L1/PD-L2/CD47*.^84–86^ Such vigilance in monitoring immunogenicity is critical for the safe and effective translation of hPSC-based therapies into clinical settings.

### When do hPSC-derived mDANs die during the transplantation process?

**Fig. 1:**
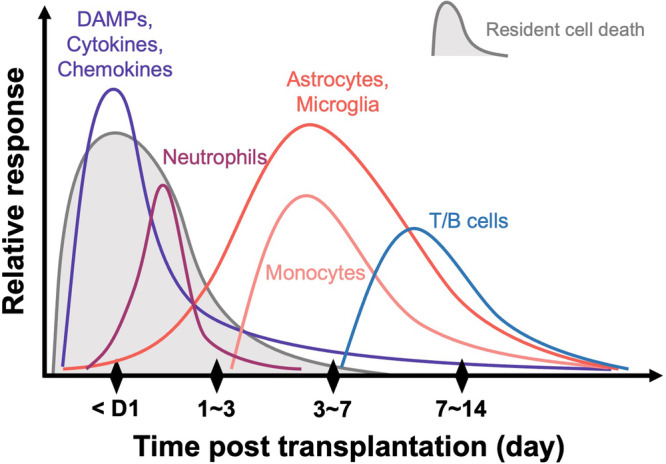
Dynamic profiles of molecular and cellular changes following needle trauma. Upon needle injection, the brain experiences physical damage, leading to the rupture of resident cells such as neurons, astrocytes, microglia, and oligodendrocytes. This rupture results from the impact of the needle, causing these cells to burst. Subsequently, the ruptured cells release damage-associated molecular patterns (DAMPs) rapidly, which affect neighboring cells and trigger the production and secretion of cytokines and chemokines due to activation. Among the cells sensing this response, neutrophils are quickly recruited to the damaged site, playing a pivotal role in promptly eliminating the debris. Concurrently, astrocytes and microglia become increasingly activated and migrate toward the damaged area over time. Approximately by the third day, peripheral monocytes infiltrate, and depending on the severity of the brain damage, T and B cells may also infiltrate, engaging in reparative functions. This sequence of inflammatory processes is crucial for the removal of cell debris resulting from needle trauma, facilitating essential steps for the repair and homeostasis of the damaged area. At the same time, however, this event appears to cause substantial damage and death to engrafted mDANs.

**Fig. 2:**
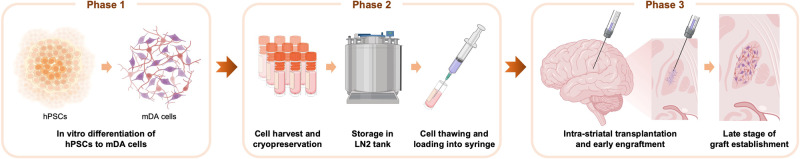
Three different phases of hPSC-based CRT when mDANs may die. Schematic representation of the three phases in which mDANs can die. Phase 1 involves the in vitro differentiation of hPSCs into mDA cells, predominantly comprising mDAPs and mDANs, using optimized procedures. Phase 2 encompasses the harvesting and cryopreservation of in vitro differentiated mDA cells, emphasizing the critical steps of cryopreservation, storage, and thawing. In Phase 3, the final in vivo transplantation occurs, consisting of surgical transplantation, the early stage (< 2 weeks), and the late stage (> 2 weeks) of graft establishment. The potential challenges and considerations at each phase, including cell viability, immune responses, and environmental factors, are discussed for a comprehensive understanding of the optimization process in hPSC-based CRT for PD.

A major advantage of hPSCs lies in their potential to produce unlimited amounts of transplantable cell sources, such as mDANs. Possibly due to this advantage, cell survival in hPSC-based CRT, unlike fVM-based CRT, has not garnered sufficient attention and remains less investigated. Given the significant differences in cellular, developmental, and proliferation properties between fVM and hPSCs, both similar and distinct factors must be considered for successful CRT in PD. To address these issues, we investigated the cell survival issue of hPSC-based CRT using several rodent models such as wild type (with immunosuppression) and athymic rats as well as immunocompromised NOD SCID gamma (NSG) mice and humanized NSG mice.^84^ Interestingly, the majority (∼90%) of mDANs (derived from both hESCs and hiPSCs) died within the first 1–2 weeks after transplantation, which is very similar to fVM transplantation.^43,46–52^ In addition, this study revealed that the transplantation procedure itself triggered an acute host inflammatory response. Remarkably, the host immune response was triggered even when only media was injected without any cell, indicating that this is induced by the host innate immune response. Because the immune response pattern was very similar to that of traumatic brain injury (TBI) (Fig. 1),^87,88^ we have termed this phenomenon “needle trauma”.^84^ This needle trauma appears to physically damage the host brain, leading to acute cell death of host neuronal cells around the injection path, which is thought to trigger subsequent immune responses such as immediate secretion of proinflammatory cytokines (e.g., TNF and IL-1β), activation of astrocytes/microglia, and robust infiltration of Iba-1^+^ and major histocompatibility complex (MHC)II^+^ inflammatory cells near the needle track, which peaked at day 7 before declining at 1 month and disappeared at 6 months. Importantly, this needle trauma preferentially led to the death of most mDANs, rather than midbrain dopamine progenitors (mDAPs), within the graft. Furthermore, unlike fVM-based CRT, the total number of mDANs (and grafted cells) significantly increased during the later stage, suggesting that some mDAPs proliferated and differentiated into new mDANs in the host brain post-transplantation.^84^ In light of these new findings, coupled with insights from previous fVM transplantation studies,^43,46–52^ we propose that hPSC-derived mDANs may die during one of three phases in the hPSC-based CRT procedure (Fig. 2).

In Phase 1, hESCs/hiPSCs are cultured and subsequently differentiated in vitro over a specific period using optimized procedures (Fig. 2). While various laboratories employ diverse optimized protocols,^65,89–96^ these methods typically use dual inhibition of SMADs, targeting BMP and TGFβ signalings, and dual activation of WNT and SHH signalings, based on previous developmental studies. This in vitro differentiation process leads to the generation of mDA cells, predominantly comprising mDAPs and mDANs, in the range of 60%–95% and 5%–25%, respectively, depending on individual protocols.^65,89–96^ These protocols vary in detailed methods and media components. For instance, some protocols utilize a 2D monolayer method, while others employ a combination of 2D and 3D cultures, such as embryoid bodies and floating neurosphere cultures. There is a possibility that mDA cells differentiated in vitro may lose viability during the differentiation process. For example, we observed that a significant portion of cells die and/or become apoptotic on evenly distributed monolayer culture and that dividing the monolayer into smaller isolated portions, known as the “spotting method”, significantly reduced the percentage of these unhealthy or apoptotic cells during in vitro differentiation process.^65,97^ Phase 1 is analogous to the initial step of fVM-based CRT, where donor embryos are dissected and prepared as either cell suspension or tissue blocks for transplantation.^43^ Previous studies demonstrated that the cell viability was substantially affected by different conditions and types of the grafting medium used for the preparation of fVM-derived cells.^98–100^ Therefore, in vitro differentiation conditions and culture media should be rigorously tested and optimized to maximize the viability. This optimization is crucial as the non-viable/apoptotic component of these final cell products will persist until transplantation and could negatively impact clinical outcomes. For instance, in conjunction with needle trauma-induced neuroinflammation, this dead or apoptotic component of transplanted cell product may trigger an additional host immune response in Phase 3. Although one can design a FACS procedure to exclude dead/dying cells, it may impose additional harmful effects on viable cells.

In Phase 2, in vitro differentiated mDA cells are harvested and cryopreserved in liquid nitrogen until transplantation (Fig. 2). Recent studies indicate that hPSC-derived mDA cells can be cryopreserved without losing their viability, cellular phenotypes, and in vivo function.^65,93,101–103^ Consequently, most groups are planning to incorporate this cryopreservation step in hPSC-based CRT. Phase 2 comprises three key steps: (i) harvesting and cryopreserving the in vitro differentiated mDA cells using an optimal cryopreservation medium in cryovials; (ii) storing multiple cryovials in liquid nitrogen; and (iii) thawing the cryovials and loading mDA cells into a surgical syringe before transplantation. In theory, mDA cells can undergo cell death at any of these steps. For example, although hPSC-derived mDA cells have been reported to maintain their viability and function, the duration for which they can be stored in liquid nitrogen without compromising their viability and function remains an area of uncertainty. Given that needle trauma has been observed to preferentially affect mDANs more than mDAPs, it is plausible that the impact of cryopreservation and subsequent handling varies significantly depending on cell types.^84^ Therefore, revisiting this issue and systematically analyzing the viability of different cell types at each step could be crucial for the final optimization of the process.

Phase 3 constitutes the final in vivo transplantation step and encompasses (i) surgical transplantation, (ii) the early stage (< 2 weeks), and (iii) the late stage (> 2 weeks) of graft establishment (Fig. 2). Host immune responses operate at these steps and may lead to the death of mDA cells. During the initial step of graft injection, mDA cells may retain viability in the syringe for a limited period (e.g., 10–20 min). However, prolonged delays during the injection process, exceeding the norm in clinical procedures, may result in the death of a portion of mDA cells (especially mDANs) even before they are injected into the host brain. Thus, minimizing delay in the surgical procedure is advisable. Notably, the surgical procedure itself appears to trigger acute innate immune responses, culminating in a hostile neuroinflammation environment. Injected mDANs are consequently subjected to early and preferential cell death. Furthermore, an adaptive immune response between grafted cells and the host immune system may occur, potentially leading to graft rejection in the absence of immunosuppression. In the later stage of engraftment in Phase 3, surviving mDA cells undergo maturation, differentiation, and integration into the host brain to establish new functional circuits. However, it is plausible that a portion of new and/or maturing mDANs may succumb even at this late stage due to various factors such as continued host immune responses, insufficient essential factors (e.g., blood supply, growth factors, and oxygen), and an unfavorable PD environment characterized by neuroinflammation, oxidative stress, and α-synucleinopathy.

## Host immune responses in HPSC-Based CRT

The survival of engrafted mDA cells is significantly influenced by various levels of host immune responses. A fundamental requirement for the survival of transplanted cells is to evade graft rejection, a process regulated by the host’s adaptive immune system. Traditionally, certain specialized organs such as the brain and the eye have been considered immune-privileged,^104^ which might explain why an earlier fVM transplantation study did not use immunosuppressants (Table 1). Conversely, an opposing viewpoint suggests that even autologous cells can elicit an immune response if differentiated cell products express immunogenic antigens.^83,105^ To investigate this contentious issue of immunogenicity in autologous vs allogeneic intracerebral grafts, we conducted transplantation experiments involving patient-derived and allogeneic mDA cells into the striatum of NSG mice, patient-humanized NSG mice, and allogeneic humanized NSG mice. Our findings revealed that while autologous mDA cells were rejected in allogeneic humanized mice, they were accepted in autologous humanized mice,^61^ indicating that allogeneic mDA cells would likely be rejected without immunosuppression when transplanted into the brain. It is noteworthy that the brain’s immune privilege may require an intact blood–brain barrier (BBB), which could be compromised in PD brains^106^ as well as by the surgical procedure. Additionally, our recent study suggests that the host immune system interacts not only with grafted cells but also with the surgical instrument, namely the needle, underscoring the critical role of the host innate immune response in the process,^84^ as described below.

### Adaptive immune response and immunosuppression

The adaptive immune response plays a pivotal role in the rejection of allogeneic or xenogeneic grafts.^107^ This adaptive immunity is characterized by the precise and sensitive reactions of T and B cells to MHCs, also known as HLA, expressed by all nucleated cells. Even slight differences in the expression of HLA molecule(s) by grafted cells can trigger active elimination by the body. Therefore, significant immunosuppression is required even for intracerebral transplantation of allogeneic mDA cells. Indeed, all current and future clinical trials involving allogeneic hPSC-derived mDA cells plan to use substantial levels of immunosuppression.^90,93,95,108^ It is crucial to acknowledge that long-term immunosuppression is associated with diverse side effects, including susceptibility to infections and malignancies, along with additional costs and inconvenience. Previous studies have demonstrated a correlation between infection risk and the dose and duration of immunosuppressants,^109,110^ with risks and severity of infection increasing when used in combination with other immunosuppressants.^111^ Additionally, immunosuppression with cyclosporine is known to elevate the risk of malignancies such as lymphoma or skin cancer.^112,113^ Other side effects may include loss of appetite, vomiting, nausea, and tremors, although these symptoms typically subside as the body adapts to immunosuppressants. Given these various side effects, researchers are actively exploring ways to optimize the administration route, dosage and duration of immunosuppression. For example, a promising strategy involves leveraging local immunosuppression to circumvent the systemic side effects associated with standard immunosuppression.^114^

### Innate immune response

Innate immunity constitutes the body’s initial line of defense, encompassing physical, chemical, and cellular mechanisms to promptly counteract or eliminate foreign antigens introduced from external sources.^107,115^ Unlike adaptive immunity, which is acquired through exposure to invaders, innate immunity is inherently present at birth and relies on antigen-nonspecific defense mechanisms. The primary function of innate immunity is to directly eliminate pathogens, and most importantly, to rapidly recruit immune cells to the site of infection and inflammation.^116,117^ This is achieved through sensing pathogens and producing cytokines (e.g., TNFα, IL-1β, IFNγ) and chemokines (e.g., CXCL, CCL). Additionally, innate immunity responds to physical damage resulting from external mechanical forces. A representative example is neuroinflammation induced by TBI, where initial traumatic insults trigger disruption of both macro-barriers (e.g., the skin) and micro-barriers (e.g., cell membranes), leading to secretion of diverse immune molecules.^118^

Our recent study showed that the transplantation procedure using a needle prominently triggers host innate immune responses, which was termed “needle trauma’ due to its similarity to TBI (Fig. 1).^84^ Primary TBI occurs immediately after the impact, damaging physical structures of the brain, including meningeal and neuronal contusion, axonal shearing, and blood vessel damage. Secondary TBI develops gradually, involving various cellular processes, such as BBB disturbance, excitotoxicity, mitochondrial dysfunction, oxidative stress, inflammation, and cell loss.^115^ Since needle trauma similarly induces these downstream pathways, further elucidation may provide promising molecular targets to mitigate its effects on the survival of grafted mDA cells, thereby potentially enhancing the outcomes of hPSC-based CRT (see below). It is worth noting that PD patients typically exhibit elevated levels of both brain inflammation and systemic inflammation compared to healthy individuals.^119–121^ This heightened inflammatory state in PD patients may potentially exert a more pronounced adverse impact on the outcome of CRT. Furthermore, it is essential to recognize that the systemic immune system varies significantly among individuals, including those with PD, which may lead to differences in treatment outcomes due to these immunological differences. However, it is important to acknowledge that the primary factor influencing the survival of transplanted mDANs is the inflammation triggered by brain damage resulting from the transplantation procedure itself. Consequently, further research efforts are needed to fully understand the distinct roles of intrinsic inflammatory states and surgery-induced inflammation in determining the survival of grafted mDANs.

## Potential strategies to enhance cell survival for successful HPSC-Based CRT

Although hPSC-based CRT offers the advantage of generating an unlimited number of transplantable cells, it also faces the challenge of poor survival hPSC-derived mDANs, as evidenced by previous pre-clinical studies (Table 2).^96,122–131^ Therefore, developing novel strategies to enhance the survival of mDANs during CRT is imperative. One promising approach is modifying the host immune responses to create a more favorable environment for the transplanted cells.

**Table 2:** List of representative preclinical studies using PSC-derived mDA cells.

Graft	Host	Application	Number of grafted cells	Number of survived TH^+^ cells		Ref.
hESC-mDA precursors	NSG mouse	N/A	150,000	4500 ± 1000		^94^
	SD rat		250,000	14,500 ± 5000		
hiPSC-NPC	NOD-SCID mouse	N/A	1 × 10^5^	7500 ± 3500 (D28 without Shh and FGF8) 2000 ± 1500 (D28 with Shh and FGF8) < 500 (D42 without Shh and FGF8) < 500 (D42 with Shh and FGF8)		^131^
	Cynomolgus monkey		2.4 × 10^6^ (right) 2.4 × 10^6^ (left)	3.07 × 10^4^ (right, d28 spheres), 1.26 × 10^5^ (left, d42 spheres)		
hESC-NPC	Cynomolgus monkey	N/A	4.8 × 10^6^	1.3–18.6 × 10^3^ in each side		^130^
PiPSC-mDANs	SD rat	N/A	100,000–200,000	2791–18,684 (sorted NCAM^+^/CD29^low^) 4835–22,666 (unsorted)		^129^
hESC-mDANs	Athymic rat	N/A	150,000	986 ± 333		^128^
hiPSC-mDAPs	SD rat	Sorting: CORIN^+^	4 × 10^5^	Unsorted: 3436 ± 2384 (D28) N/A (D42)	CORIN^+^ sorted: 6747 ± 2341 (D28) 1900 ± 658 (D42)	^127^
CM-iPSC-mDANs	Cynomolgus monkey	N/A	MF25–04 : 1 × 10^7^	13,029 (MF25–04)		^134^
			MF27-04, MF66-02 : 4 × 10^7^	8551 (MF27-04), 7938 (MF66-02)		
hESC-mDANs	SCID mouse	N/A	2 × 10^5^	7555 ± 913 (hM4Di-), 7245 ± 517 (hM3Dq-), 6110 ± 254 (EGFP-expressing cell)		^126^
hESC/hiPSC-mDAPs	SD rat	Sorting: LRTM1^+^	1.3 × 10^5^	Unsorted: 1102 ± 349	LRTM1^+^ sorted: 11,702 ± 2566	^125^
	Cynomolgus monkey		1 × 10^6^	N/A	LRTM1^+^ sorted: ~2.4 × 10^6^	
hiPSC-mDAPs	Cynomolgus monkey	N/A	4.8 × 10^6^	Healthy : 5.4 ± 4.7 × 10^4^ PD : 7.3 ± 5.3 × 10^4^		^124^
hESC-mDAPs	Fischer 344 rat	Encapsulation: HA-heparin-RGD hydrogel	89,500 (2D)	2D, Suspension: ∼1200		^170^
			93,800 (3D)	3D, HA hydrogel: ∼6400		
hiPSC-mDAPs	Athymic rat	N/A	100,000	5621 ± 1029 per 100,000		^65^
hESC-PITX3/LMX1A-GFP VM mDAPs	Athymic rat	AAV-GDNF	100,000	PITX3-GFP^+^ cells: 4092 ± 602	+ GDNF: PITX3-GFP^+^ cells 7986 ± 1375	^123^
				LMX1A-GFP^+^ cells: 19,810 ± 2828	+ GDNF: LMX1A-GFP^+^ cells 30,381 ± 6513	
hiPSC-mDAPs	F344-Il2rgem2Kyo X-SCID rat	Zonisamide	5 × 10^5^	3.17 ± 1.64 × 10^3^ (vehicle) 7.5 ± 1.95 × 10^3^ (+ZNS 30 mg/kg) 8.16 ± 3.43 × 10^3^ (+ZNS 60 mg/kg)		^122^
hiPSC-mDAPs	F344 Njc1-rnu/rnu rat	N/A	4 × 10^5^	2835 ± 2534		^90^
hESC-mDAPs	Athymic rat	N/A	400,000	49,250 (male), 42,480 (female)		^93^
hiPSC-mDA neurospheres	F344 Njc1-rnu/rnu rat	N/A	400,000 (fresh) 400,000 (cryopreservedX1) 800,000 (cryopreservedX2)	4601 ± 1189 (fresh) 1306 ± 480 (cryopreservedX1) 3988 ± 1961 (cryopreservedX2)		^102^
hiPSC-PITX3-GFP mDAPs	Athymic rat	AAV-GDNF	100,000	18,910 ± 1853 (Homotopic) 20,550 ± 1616 (Ectopic)	+ GDNF: 28,088 ± 2618 (Homotopic) 24,396 ± 1180 (Ecotopic)	^177^
hiPSC-mDAPs	NSG mouse	T_REG_	100,000	+ Saline: 1825 ± 287 (2 weeks) 5421 ± 534 (20 weeks)	+ T_REG_ : 4427 ± 189 (2 weeks) 9523 ± 835 (20 weeks)	^84^
hESC-mDAPs	Athymic rat	N/A	30,000	∼1900		^95^
			60,000	∼3700		
			120,000	∼8000		
hESC-mDAPs	Athymic rat	N/A	5000	758.86 ± 286.00		^96^
			10,000	1477.71 ± 529.79		
			25,000	2820.00 ± 1378.86		
			100,000	17,598.40 ± 5451.54		

*hESC* human embryonic stem cell, *mDA* midbrain dopaminergic, *NSG* NOD SCID gamma, *SD* Sprague Dawley, *hiPSC* human induced pluripotent stem cell, *NPC* neural progenitor cell, *NOD* nonobese diabetic, *SCID* severe combined immunodeficient, *Shh* sonic hedgehog, *FGF8* fibroblast growth factor 8, *PiPSC* primate induced pluripotent stem cell, *mDANs* midbrain dopaminergic neurons, *NCAM* neural cell adhesion molecule, *CM* Cynomolgus monkey, *mDAPs* midbrain dopaminergic progenitor cells, *PD* Parkinson’s disease, *ZNS* Zonisamide, *GDNF* Glial cell line-derived neurotrophic factor, *RGD* Arginylglycylaspartic acid, *HA* Hyaluronic acid, *T*_*REG*_ Regulatory T cells, *Ref.* reference.

### Strategies targeting adaptive immunity

**Fig. 3:**
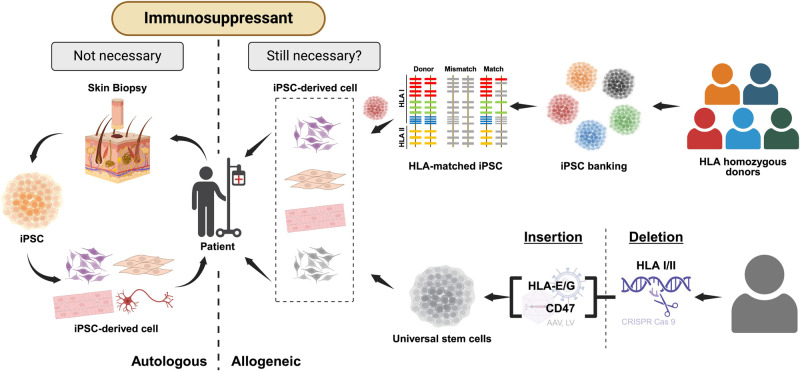
Strategies targeting adaptive immunity. In general, allogeneic transplantation using mDA cells derived from hESCs still necessitates immunosuppression, whereas autologous transplantation with mDA cells from hiPSCs is characterized by immune tolerance, eliminating the need for immunosuppressants. An alternative strategy, distinct from utilizing autologous cells, involves the application of HLA-matched hiPSCs to reduce the risk of graft rejection, with ongoing efforts to establish an HLA-matched iPSC bank encompassing a diverse array of donors. Another approach focuses on the development of "universal donor stem cells," incorporating genetic modifications, such as CRISPR/Cas9-mediated knockout of HLA class I and II components, along with lentiviral overexpression of the immune receptor CD47 or HLA-E/G transgene.

A prerequisite for the survival of grafted cells is to circumvent graft rejection. Consequently, all allogeneic transplantations require a significant level of immunosuppression, although the duration of immunosuppression required for each patient remains uncertain. Recent studies in nonhuman primates^132–134^ and humans^61^ have demonstrated that autologous transplantations do not require immunosuppression. However, this autologous approach entails substantial time and expense. An alternative strategy to mitigate the need for immunosuppression involves using HLA-matched iPSCs. Supporting evidence for this approach comes from a recent primate study indicating that transplantation of mDA cells from HLA-matched primate iPSCs reduced host immune responses and increased the survival of mDANs.^12^ Consequently, numerous groups are striving to establish HLA-matched hiPSCs as a bank from common HLA-homozygous donors, aiming to minimize graft rejection post-transplantation and reduce the time and effort compared to the autologous approach (Fig. 3).^135,136^ Yamanaka’s group estimated that hiPSC lines derived from approximately 140 unique HLA-homozygous donors would be sufficient to cover up to 90% of the Japanese population.^137^ Due to genetic diversity, large-scale hPSC banks are currently being established in the United States to cover diverse ethnic groups such as European Americans, African Americans, Hispanics, and Asians.^138^ Despite these efforts, the immune response may still occur even with HLA-matched cells due to indirect pathway caused by H-Y minor histocompatibility antigens or innate immunity resulting from natural killer (NK) cells.^139,140^

Efforts to develop “universal donor stem cells” that evade immune rejection have garnered significant attention.^141,142^ This approach primarily aims to eliminate HLA molecules in donor cells, which are pivotal for adaptive immunity and graft rejection. The initial endeavor aimed to eliminate HLA molecules, specifically targeting HLA class I, which predominantly expresses β2-microtubulin (B2M), a structurally non-polymorphic heavy chain, across most cell types.^143,144^ However, cells lacking HLA class I, generated through the genomic engineering approach, exhibited limitations as they were susceptible to lysis by NK cells via a ‘missing self’ response.^145^ Notably, the lysis mechanism is averted when the CD94/NGK2A complex on NK cells engages with any HLA class I molecule, including HLA-E, recognized for its minimal polymorphism and expression solely in B2M knockout cells.^145,146^ Recently, through the process of technology development and optimization, Schrepfer and colleagues^147^ devised a method to generate hypoimmunogenic donor iPSCs through three steps: (1) CRISPR/Cas9-mediated knockout of the *B2M* gene (a component of HLA class I), and (2) the *CIITA* gene (the master regulator of HLA class II), and (3) the lentiviral overexpression of the immune receptor CD47 transgene. They demonstrated that various cell types derived from these engineered donor iPSCs (e.g., endothelial cells, smooth muscle cells, and cardiomyocytes) effectively evade immune rejection in fully HLA-mismatched host animals and survive long-term without immunosuppression. Additionally, Hotta and colleagues created pseudo-homozygous iPSC lines by disrupting *HLA-A* and *HLA-B* bi-allelically while retaining a single *HLA-C* allele using CRISPR-Cas9.^148^ It is estimated that 12 HLA-C-retained iPSC lines with HLA-class II knockout could be immunologically compatible with > 90% of the world’s population. While these strategies hold promise for iPSC-based regenerative medicine applications, it remains uncertain whether these engineered cells would retain full functionality and hypoimmunogenicity post-transplantation into patients.^149^ Extensive genetic manipulation (e.g., ectopic CD47 expression) may lead to unforeseen adverse effects like oncogenic transformation and compromised immune responses.^150,151^ Moreover, in instances where a cell becomes infected with a virus, prompt removal may not be feasible, prompting the need for the development of genetic integration of kill-switches. Taken together, this strategy could potentially induce unintended adverse reactions, necessitating further research to refine and implement safety strategies.^152,153^ Furthermore, even if these genetic alterations successfully evade immune rejection by adaptive immunity, the transplanted grafts may still be susceptible to innate immune responses by needle trauma.

### Strategies targeting innate immunity

**Fig. 4:**
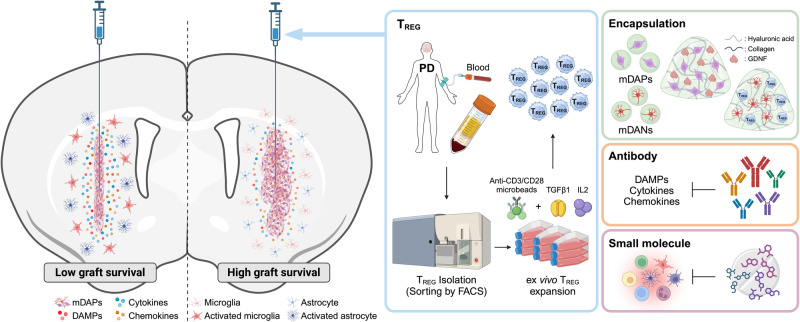
Strategies targeting innate immunity. In CRT, the standard procedure involves the injection of cells into the brain using a needle, which induces needle trauma and subsequent secretion of various innate immune response factors, including DAMPs, pro-/anti-inflammatory cytokines, and chemokines. These factors activate surrounding glial cells and lead to the infiltration of peripheral immune cells into the brain, contributing positively to damage repair. However, they also exert a detrimental effect on grafted cells, resulting in severe cell death of mDANs. A potential strategy to enhance the survival of grafted cells involves obtaining autologous T_REG_ cells from the patient, increasing their quantity and functionality, and confirming improved therapeutic effects through co-transplantation into the brain. Additionally, exploring the efficacy of inhibitors, neutralizing antibodies, and encapsulation methods to enhance the survival of grafted cells within the inflammatory microenvironment, combined with the application of T_REG_ technology, represents a promising direction for future advancements in CRT.

The host innate immune response triggered by TBI or needle trauma initiates acute inflammation after injury. This response is characterized by the secretion and upregulation of DAMPs, cytokines, chemokines, immune cell infiltration (e.g., neutrophils and myeloid cells), and subsequent activation of glial cells (astrocyte and microglia) and recruitment of leukocytes.^107,115,154^ Numerous studies have demonstrated that these molecules including DAMPs, cytokines, and chemokines, are acutely secreted after TBI within 6 h.^155^ Consequently, various research endeavors have sought to target innate immunity for therapeutic development in TBI. These investigations have revealed that blocking those molecules using specific inhibitors resulted in prominent effects to treat TBI in animal models.^156–159^ It will be intriguing to determine whether these inhibitors and/or neutralizing antibodies can produce similar effects in needle trauma and improve the survival of engrafted mDA cells. This avenue warrants future investigation (Fig. 4). Moreover, considering that needle trauma acutely triggers the secretion of various cytokines, chemokines, and DAMPs (within minutes to hours post-injury), an intriguing approach could involve delaying the grafting of cells post-needle insertion (without cells). Indeed, previous studies have shown that neuronal survival markedly increased when the injection of dopaminergic cell suspension was delayed for more than one hour following cannula insertion.^160,161^

Regulatory T (T_REG_) cells play a vital role in maintaining immunological tolerance and homeostasis.^162,163^ They are implicated in numerous autoimmune and inflammatory diseases and have been clinically utilized to improve survival rates in various organ transplantations through adoptive transfer after ex vivo expansion.^164^ In TBI, T_REG_ cells infiltrate the damaged area following initial inflammatory cell infiltration to facilitate the repair process.^165,166^ Therefore, it was hypothesized that autologous T_REG_ cells might mitigate the innate immune response triggered by needle trauma and enhance the survival of grafted mDANs. In testing the effect of T_REG_ cells via conventional adoptive transfer,^164,167^ approximately 1,000,000 ex vivo expanded autologous T_REG_ cells, were intravenously injected, leading to a modest but significant reduction in the innate immune response, as indicated by decreased infiltration of MHCII^+^ cells. However, due to the rapid onset of needle trauma-induced neuroinflammation in the brain, the adoptive transfer method may be inefficient as it requires time for the T_REG_ cells to infiltrate and function. Direct intra-striatal transplantation of only 2% (20,000) T_REG_ cells, on the other hand, led to robust suppression of needle trauma-induced inflammation and significantly increased the survival of grafted mDANs (Table 2), accompanied by faster and more pronounced behavioral improvement in rodent models of PD.^84^ Nevertheless, the rescue of mDANs was incomplete, suggesting that further optimization of intra-striatal co-transplantation of T_REG_ cells is necessary. One potential approach could involve encapsulating T_REG_ and mDA cells using hydrogel systems before transplantation (Fig. 4).^168–170^ Additionally, considering that needle trauma may occur in CRT for other CNS and non-CNS diseases, it is of significant interest to test whether T_REG_ cell co-transplantation can similarly reduce needle trauma-induced inflammation and enhance the survival of desired therapeutic cell products in general.^171^

### Strategies targeting long-term differentiation and maturation at the later stage of graft establishment

Even if grafted mDA cells manage to evade the initial immune attacks post-transplantation and survive the early stage, they face a suboptimal environment unlike early brain development, where all necessary nutrients, oxygen, growth factors, relevant transcription factors, and developmental signals are provided or induced in a precise temporal and spatial manner. Instead, the new environment of grafted mDA cells is characterized by aged PD pathological conditions such as elevated neuroinflammation, oxidative stress, α-synucleinopathy, and limited/no supply of essential factors.^172,173^ Indeed, recent single-cell RNA-seq analyses have revealed that grafted cells often exhibit inadequate and inefficient differentiation into mDANs, favoring instead the differentiation into alternative cell types such as astrocytes and vascular leptomeningeal cells.^174^ These inadequate and heterogeneous graft cell populations may be non-functional and could potentially induce unwanted immunogenicity. This challenge is compounded by the lengthy process for these mDA cells to mature into mDANs with neuronal outgrowth, reinnervate to host brain, and eventually establish functional new networks. This process can take considerable time ranging from 4–6 months in rodent brains to 1–3 years in human brains (Table 1). Therefore, it is crucial to develop and implement therapeutic strategies aimed at enhancing the survival, differentiation, and/or maturation of grafted mDA cells. Supporting this idea, pre-treatment of fVM cells with growth factors such as bFGF and GDNF increased the survival of grafted mDANs about 2-fold,^53,54^ while long-term supply of bFGF led to a 10-fold increase in the number of survived mDANs.^55^ Furthermore, recent studies have demonstrated that pretreatment or viral delivery of GDNF enhanced the survival and differentiation of hPSCs (Table 2).^123,175–177^ Building upon these promising findings, a potential strategy involves facilitating the sustained supply of relevant growth factor(s) and/or transcription factor(s), which may not only enhance the survival of grafted mDA cells but also promote their differentiation into new mDANs.

## Conclusions and perspective

Since the hallmark pathological feature of PD is the selective loss of mDANs in the substantia nigra, CRT has been the focus of extensive studies for over four decades. In particular, fVM-based CRT has provided not only proof-of-concept but also invaluable lessons. Among these, the most significant lesson is that the survival of implanted mDANs is very limited, potentially underlying the variable and often inefficient clinical outcomes. Despite significant research efforts, the molecular and cellular mechanisms underlying the acute and extensive death of mDANs post-transplantation remain only partially understood.

Recent advancements in stem cell technology offer promise for hPSC-based CRT, with various hPSC sources (e.g., allogeneic hESCs, allogeneic hiPSCs, HLA-matched hiPSCs, and autologous hiPSCs) being explored for scalable production of transplantable mDA cells in ongoing or upcoming clinical trials. However, several critical issues must be addressed for the successful implementation of hPSC-based CRT. Firstly, improving the survival of implanted mDANs is paramount, drawing from lessons learned from fVM-based CRT. Secondly, addressing the multiple phases and steps of CRT where mDANs are at risk of dying is essential. Thirdly, understanding and manipulating both host adaptive and innate immune responses are crucial, with recent evidence highlighting the role of the surgical procedure in triggering host innate immune responses. Future research efforts should focus on developing effective strategies to manipulate host immune responses to enhance the survival of implanted mDANs and improve clinical outcomes.

It is important to note that, although grafted mDANs may survive post-transplantation, they face the unfavorable PD host environment including neuroinflammation, α-synucleinopathy, and limited supply of essential factors. These challenges make their prolonged survival and maturation very difficult, necessitating further research in this aspect. Additionally, PD patients often exhibit degeneration of other neurons beyond mDANs, such as noradrenergic and/or serotonergic neurons,^178–181^ contributing to various non-motor deficits. Therefore, even the most successful hPSC-based CRT may not provide a ‘cure’ for PD. Instead, it is expected to be an integral component of a comprehensive treatment strategy, complementing other approaches such as novel drug treatment (e.g., anti-inflammatory and neuroprotective) and gene therapy.
